# Memory deficits in children and adolescents with psychotic disorders: A systematic review and meta-analysis

**DOI:** 10.1192/j.eurpsy.2023.728

**Published:** 2023-07-19

**Authors:** E. Rodríguez Toscano, P. Díaz-Carracedo, P. de la Higuera-González, G. Padilla, A. de la Torre-Luque

**Affiliations:** 1 Experimental Psychology, Cognitive Processes and Speech Therapy Department; 2 Universidad Complutense Madrid, Madrid, Spain

## Abstract

**Introduction:**

Cognitive symptoms in psychosis represent a major unmet clinical need (Acuna-Vargas et al. Cog in Psych 2019; 21(3), 223–224). Deficit in memory has been largely described in first episode early onset psychosis (Mayoral et al. Eur Psych 2008; 23(5), 375-383) and has been associated to a worse functionality (Øie et al. Neuropsychology 2011; 25(1), 25–35). However, results from existing studies are quite mixed on memory deficits of early psychosis patients, particularly in terms of memory contents and storage resources.

**Objectives:**

The aims of this study were 1) to examine the nature and extent of cognitive impairment in early-onset psychosis and 2) to analyze which type of memory (verbal and visual) is more affected in the disorder.

**Methods:**

The present systematic review and meta-analysis was conducted according to the PRISMA criteria (Moher et al. Systematic Reviews 2015; 4(1), 1 - 9). A systematic search of CINAHL, PsycInfo, PubMed, Redalyc, SCOPUS and Web of Science (published from 2000 to 2020) identified case-control studies of early onset psychotic disorder (under 18 years old). Those studies focused on both verbal and visual memory performance.

**Results:**

Twenty articles were included in the review. A deficit in memory in child and adolescent psychotic disorders was obtained displaying a large effect size in memory tasks (g = -0.83). Also, a medium effect size was found in visual memory tasks (g = - 0.61) and a large effect size was found in verbal memory tasks (g = -1.00).

**Conclusions:**

It was observed a strong memory deficit on early psychotic disorders already present at the onset of the illness. This deficit was stronger when verbal memory tasks were used compared to the effect found with visual memory tasks. Based on previous literature (García-Nieto et al. Jou Cli Child & Ado Psych 2011; 40(2), 266-280; Lepage et al. Eur Psych 2008; 23(5):368- 74; Hui et al. Psych Med 2016; 46(11):2435-44), these results contribute to describe and characterize the cognitive symptoms in the first-episode psychosis in a youth population.

**Disclosure of Interest:**

None Declared

